# Enhancing poly-γ-glutamic acid production in *Bacillus amyloliquefaciens* by introducing the glutamate synthesis features from *Corynebacterium glutamicum*

**DOI:** 10.1186/s12934-017-0704-y

**Published:** 2017-05-22

**Authors:** Jun Feng, Yufen Quan, Yanyan Gu, Fenghong Liu, Xiaozhong Huang, Haosheng Shen, Yulei Dang, Mingfeng Cao, Weixia Gao, Xiaoyun Lu, Yi Wang, Cunjiang Song, Shufang Wang

**Affiliations:** 1 Key Laboratory of Molecular Microbiology and Technology for Ministry of Education, Nankai University, Tianjin, 300071 China; 2 Key Laboratory of Biomedical Information Engineering of Ministry of Education, School of Life Science and Technology, Xi’an Jiaotong University, Xi’an, 710049 Shaanxi China; 3 Department of Biosystems Engineering, Auburn University, Auburn, AL 36849 USA; 4 State Key Laboratory of Medicinal Chemical Biology, Nankai University, 94 Weijin Road, Tianjin, 300071 China; 5 Department of Chemical and Biological Engineering, Iowa State University, Ames, IA 50011 USA

**Keywords:** Poly-γ-glutamic acid, NADPH-dependent glutamate dehydrogenase, Metabolic toggle switch

## Abstract

**Background:**

Poly-γ-glutamic acid (γ-PGA) is a valuable polymer with glutamate as its sole precursor. Enhancement of the intracellular glutamate synthesis is a very important strategy for the improvement of γ-PGA production, especially for those glutamate-independent γ-PGA producing strains. *Corynebacterium glutamicum* has long been used for industrial glutamate production and it exhibits some unique features for glutamate synthesis; therefore introduction of these metabolic characters into the γ-PGA producing strain might lead to increased intracellular glutamate availability, and thus ultimate γ-PGA production.

**Results:**

In this study, the unique glutamate synthesis features from *C. glutamicum* was introduced into the glutamate-independent γ-PGA producing *Bacillus amyloliquefaciens* NK-1 strain. After introducing the energy-saving NADPH-dependent glutamate dehydrogenase (NADPH-GDH) pathway, the NK-1 (pHT315-gdh) strain showed slightly increase (by 9.1%) in γ-PGA production. Moreover, an optimized metabolic toggle switch for controlling the expression of ɑ-oxoglutarate dehydrogenase complex (ODHC) was introduced into the NK-1 strain, because it was previously shown that the ODHC in *C. glutamicum* was completely inhibited when glutamate was actively produced. The obtained NK-PO1 (pHT01-xylR) strain showed 66.2% higher γ-PGA production than the NK-1 strain. However, the further combination of these two strategies (introducing both NADPH-GDH pathway and the metabolic toggle switch) did not lead to further increase of γ-PGA production but rather the resultant γ-PGA production was even lower than that in the NK-1 strain.

**Conclusions:**

We proposed new metabolic engineering strategies to improve the γ-PGA production in *B. amyloliquefaciens*. The NK-1 (pHT315-gdh) strain with the introduction of NADPH-GDH pathway showed 9.1% improvement in γ-PGA production. The NK-PO1 (pHT01-xylR) strain with the introduction of a metabolic toggle switch for controlling the expression of ODHC showed 66.2% higher γ-PGA production than the NK-1 strain. This work proposed a new strategy for improving the target product in microbial cell factories.

**Electronic supplementary material:**

The online version of this article (doi:10.1186/s12934-017-0704-y) contains supplementary material, which is available to authorized users.

## Background

Poly-γ-glutamic acid (γ-PGA) is a valuable polymer consisting of d/l-glutamate monomers with the peptide bond formed between the α-amino group and the γ-carboxyl group [1, 2]. It has many favorable features such as being biodegradable, water soluble, edible and non-toxic to humans and the environment, and has been widely used for various applications in food, medicine, cosmetic and agriculture [3]. γ-PGA-producing strains are classified as either glutamate-dependent ones or glutamate-independent ones [3]. Glutamate-independent strains are preferable for industrial γ-PGA production because of their low cost and simple fermentation process [4]. We previously isolated a glutamate-independent γ-PGA producing strain *Bacillus amyloliquefaciens* LL3 from the fermented food, and it can produce γ-PGA efficiently by using sucrose and ammonium sulfate as substrate [4]. In this work, we aimed to explore new metabolic engineering strategies to further improve γ-PGA production based on this host strain.

**Fig. 1:**
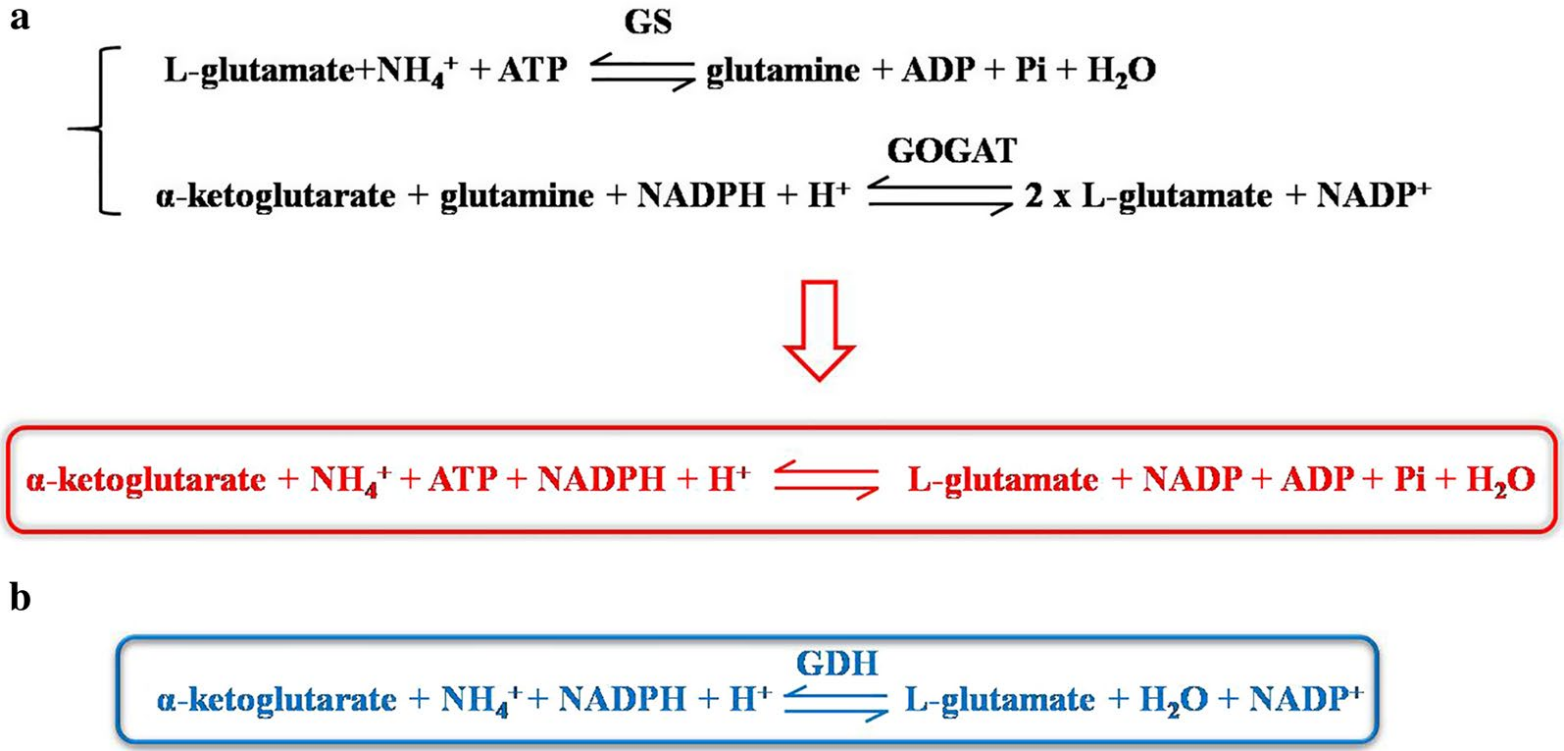
Comparison of the two glutamate biosynthetic pathways existing in nature. **a** GS–GOGAT pathway; **b** NADPH-dependent glutamate dehydrogenase (GDH) pathway

**Fig. 2:**
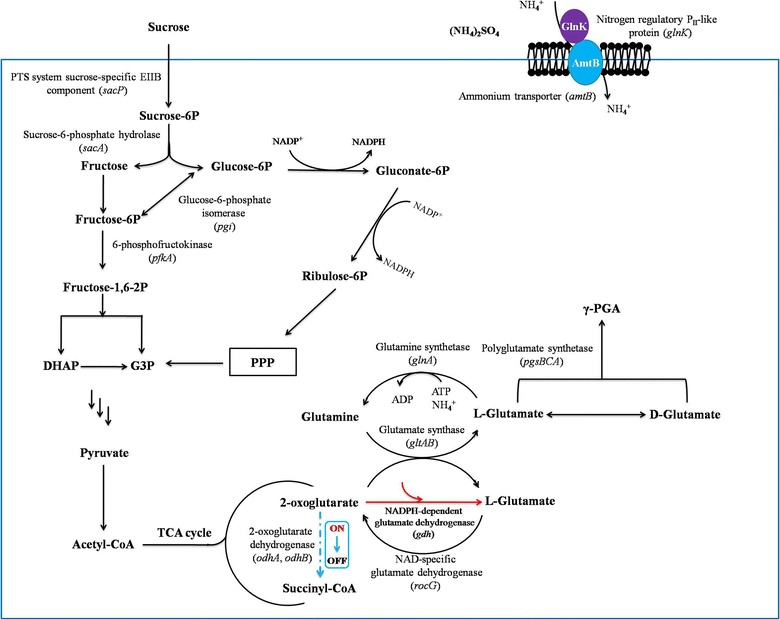
γ-PGA synthesis pathway in *Bacillus amyloliquefacien*s NK-1 and the schematic of the metabolic engineering work carried out in this study

Intracellularly, γ-PGA is synthesized by γ-PGA synthetase PgsBCA with glutamate as its sole precursor. The precursor glutamate can be obtained from extracellular supplement or intracellular synthesis from ɑ-oxogluterate. Glutamate-independent γ-PGA producing strain can use inorganic nitrogen sources to synthesize glutamate for γ-PGA production. The intracellular glutamate synthesis capability is a limiting factor for γ-PGA synthesis and thus the improvement of intracellular glutamate synthesis might also lead to the improvement of the γ-PGA production. *Corynebacterium glutamicum* is well known as a workhorse for glutamate production from long time ago [5, 6]. It has excellent features for glutamate synthesis, and can generate large amount of glutamate under desirable culture conditions [7]. There are two glutamate synthesis pathways in *C. glutamicum*. Besides the GS-GOGAT pathway (Glutamine synthetase-glutamate synthase pathway), *C. glutamicum* has a NADPH-dependent GDH (glutamate dehydrogenase) glutamate synthesis pathway [8]. The NADPH-dependent GDH pathway seems to be energy-saving when compared with the GS-GOGAT pathway because it does not need ATP for the glutamate synthesis (Fig. 1). However, *B. amyloliquefaciens* synthesizes glutamate via the GS-GOGAT pathway exclusively. Although a GDH exists in *B. amyloliquefaciens*, it is NAD-dependent, and responsible for glutamate degradation rather than production. ɑ-oxoglutarate is an essential precursor for glutamate synthesis; meanwhile it also serves as the substrate of ɑ-oxoglutarate dehydrogenase complex (ODHC) for succinyl-CoA synthesis in a competing pathway for glutamate production (Fig. 2). Previous work found that the ODHC activity was not detectable when *C. glutamicum* actively produced glutamate [9]. Therefore, in this study, we aimed to introduce these two unique metabolic features from *C. glutamicum* into *B. amyloliquefaciens* NK-1 to boost its glutamate and thus γ-PGA production. Firstly, we introduced the NADPH-dependent GDH gene (*gdh*) from *C. glutamicum* ATCC13032 to the NK-1 strain by plasmid-based overexpression, and the γ-PGA production was improved by 9.1% in the mutant compared to the NK-1 host strain. Secondly, we constructed a metabolic toggle switch in the NK-1 strain to control the expression of *odhAB* (encoding ɑ-oxoglutarate dehydrogenase complex responsible for the succinyl-CoA synthesis). This led to a 66.2% increase of γ-PGA production. To our best knowledge, this study is the first report about improving γ-PGA production by integrating the *C. glutamicum* glutamate synthesis features into the γ-PGA producing strain. This work provides valuable references for relevant researchers who work on biochemical production through metabolic engineering strategies.

## Methods

### Microorganisms, plasmids and cultivation conditions

Strains and plasmids used in this work are listed in Table 1. *C. glutamicum* ATCC13032 were purchased from China General Microbiological Culture Collection Center (CGMCC). All of the *B. amyloliquefaciens*, *C. glutamicum* and *Escherichia coli* strains were grown at 37 °C in Luria–Bertani (LB) medium for routine strain construction and maintenance. *B. amyloliquefaciens* was cultured in γ-PGA fermentation medium for γ-PGA production following our previously reported protocols [10]. γ-PGA was purified and weighed following a previously described method [11]. When required, antibiotics were used at the following concentrations: 100 μg/mL ampicillin, 5 μg/mL chloramphenicol, 5 µg/mL erythromycin. The concentration of 5-fluorouracil used for mutant strain selection was 100 μg/mL.

**Table 1:** Strains and plasmids used in this study

Strains and plasmids	Relevant genotype and characteristics	Source
Strains		
*B. amyloliquefaciens* NK-1	LL3 derivative, ΔpMC1, Δ*upp*	[12]
*B. amyloliquefaciens* NK-1 (pHT315-gdh)	*B. amyloliquefaciens* NK-1 with the expression plasmid pHT315-gdh	This work
*B. amyloliquefaciens* NK-1 (pHT315-cgdh)	*B. amyloliquefaciens* NK-1 with the expression plasmid pHT315-cgdh	This work
*B. amyloliquefaciens* NK-1 (pHT01 + pCB-P_xyl_)	NK-1 derivative with the plasmids pHT01 and pCB-P_xyl_	This work
*B. amyloliquefaciens* NK-1 (pHT01-xylR + pCB-P_xyl_)	NK-1 derivative with the expression plasmids pHT01-xylR and pCB-P_xyl_	This work
*B. amyloliquefaciens* NK-TP	NK-1 derivative with its native *odhA* promoter replaced by the P_xyl_ promoter	This work
*B. amyloliquefaciens* NK-TP (pHT01-xylR)	NK-TP derivative with the expression plasmid pHT01-xylR	This work
*B. amyloliquefaciens* NK-PO1	NK-1 derivative with its native *odhA* promoter replaced by the PO1 promoter	This work
*B. amyloliquefaciens* NK-PO1 (pHT01-xylR)	NK-PO1 derivative with the expression plasmid pHT01-xylR	This work
*B. amyloliquefaciens* NK-PO1 (pHT01-xylR + pHT315-gdh)	NK-PO1 derivative with the expression plasmids pHT01-xylR and pHT315-gdh	This work
*B. amyloliquefaciens* NK-PO1 (pHT01-xylR + pHT315-cgdh)	NK-PO1 derivative with the expression plasmids pHT01-xylR and pHT315-cgdh	This work
*C. glutamicum* ATCC13032	Glutamic acid producing strain	Lab stock
*E. coli* DH5α	F^−^, φ80d*lac*ZΔM1, Δ(*lacZYA*-*argF*)U169, *deoR*, *recA*1, *endA*1, *hsdR*17(r_k_^−^, m_k_^+^), *phoA*, *supE*44, λ^−^ *thi*-1, *gyrA*96, *relA*1	Lab stock
*E. coli* GM2163	F^−^, *ara*-*14 leuB6 thi*-*1 fhuA31 lacY1 tsx*-*78 galK2 galT22 supE44 hisG4 rpsL 136 (Str* ^*r*^ *) xyl*-*5 mtl*-*1 dam13::*Tn9 (Cam^r^) *dcm*-*6 mcrB1 hsdR2 mcrA*	Lab stock
Plasmids		
pKSU	pKSV7 derivation with *upp* gene expression cassette	[15]
pHT01	Cm^r^, IPTG inducible expression vector for *Bacillus*	MoBiTec
pHT315	Em^r^, IPTG inducible expression vector for *Bacillus*	[32]
pCB	pHT315 derivation with the *bgaB* expression cassette	[13]
pKSV7-P_xyl_	p-KSU derivation with insertion fragment P_xyl_	This work
pHT01-xylR	pHT01 derivative with the *xylR* gene	This work
pCB-P_xyl_	pCB derivation with the P_xyl_ promoter upstream the *bgaB* gene	Lab stock
pHT315-gdh	pHT315 derivation with the condon optimized *gdh* gene	This work
pHT315-cgdh	pHT315 derivation with the *gdh* gene from *C. glutamicum* ATCC13032	This work

### DNA manipulation, plasmid construction and strain development

The gene insertion plasmids pKSV7-P_xyl_ and pKSV7-PO1 were constructed following the previously reported procedures [12]. The NADPH-dependent glutamate dehydrogenase gene from *C. glutamicum* ATCC13032 was codon optimized to match the *Bacillus.* sp codon usage and commercially synthesized by Genescript (Nanjing, China). The codon optimzed *gdh* gene and the native glutamate dehydrogenase gene (*cgdh*) were put downstream of the P_43_ promoter and ligated into the pHT315 plasmid using the restriction enzyme sites of *Eco*RI and *Xba*I, generating plasmids pHT315-gdh and pHT315-cgdh. The *xylR* gene was put downstream of the P_grac_ promoter and ligated into the *Bam*HI site of pHT01, generating plasmid pHT01-xylR. The P_xyl_ promoter (xylose inducible promoter from pWH1520) was put upstream of the *bgaB* gene and ligated together into the *Kpn*I and *Sal*I sites of pCB [13], generating plasmid pCB-P_xyl_.

To control the *odhAB* gene expression, the P_xyl_ promoter or the PO1 promoter was inserted individually into the upstream of the *odhA* gene in NK-1 strain, by a marker-less gene manipulation method [14, 15]. To avoid the leaky expression from its native P_odhAB_ promoter, the *B. subtilis spoVG* gene transcription terminator was integrated into the chromosome downstream the P_odhAB_ promoter and upstream the newly inserted promoter (either P_xyl_ or PO1) [16]. The obtained strains were designated as *B. amyloliquefaciens* NK-TP and *B. amyloliquefaciens* NK-PO1, respectively.

As a naming rule in this study, the plasmid N was transformed into the *B. amyloliquefaciens* X strain and the resultant strain was designated as *B. amyloliquefaciens* X (N) (N represents the plasmid name and X represents the corresponding strain name). All the primers used in this work are listed in Additional file 1: Table S1. The sequences of relative genes and genetic elements are listed in Additional file 2.

### β-Galactosidase activity assays

A reporter gene *bgaB* was used to verify the activity of the metabolic toggle switch. The β-galactosidase activity in the cell culture broth of NK-1 (pHT01 + pCB-P_xyl_) and NK-1 (pHT01-xylR + pCB-P_xyl_) was determined every 6 after 12 h of cultivation according to the previously described method [17]. 0.15 mL bacterial culture was mixed with 0.375 mL Z buffer (60 mM Na_2_HPO_4_, 40 mM NaH_2_PO_4_, 10 mM KCl, 1 mM MgSO_4_, 50 mM β-mercaptoethanol, pH 7.0) and 0.75 mL lysozyme (4 mg/mL lysozyme dissolved in Z buffer) and the mixture was incubated at 37 °C for 30 min. 6 μL of 10% Triton X-100 was then added, vortexed briefly and pre-warmed for 3–5 min in a 55 °C water bath prior to adding 0.15 mL ONPG (o-nitrophenyl-β-d-galactopyranoside) (4.0 mg/mL in Z buffer). The reaction time was recorded and the reaction was stopped by adding 0.3 mL Na_2_CO_3_ solution (1 M). Samples were centrifuged for 10 min and the absorbance of the supernatant was measured at 420 nm (A420). The result reported in this study was the average from three reactions. One unit of β-galactosidase activity was defined as the amount of enzyme that resulted in one absorbance change (at 420 nm) per minute under 1 mL assay condition.

### NADPH-GDH activity assays

The NADPH-GDH activity was measured as described previously [18]. Cells were cultivated in 100 mL of γ-PGA fermentation medium in 500 mL flasks for 36 h. The cell pellets were washed twice by potassium phosphate buffer (pH 6.9) and then resuspended in 10 ml of the same buffer. Cells were broken with sonication (600 W for 30 min with cycles of 3 s sonication followed by 3 s pause). The broken cells were centrifuged at 12,000 rpm for 3 min and the supernatants were used for the NADPH-GDH activity measurement. To measure the NADPH-GDH activity, the reaction mixture (containing 100 μL of 1 M Tris–HCl, pH 8.0; 100 μL of 2.5 mM NADPH; 100 μL of 200 mM NH_4_Cl; 100 μL cell extract) was added to an UV-cuvette. The reaction was started by the addition of 100 μL of 100 mM 2-oxoglutarate and the absorbance decrease at 340 nm was measured using the UVIS 200 detector (Alltech, USA). One unit (U) of the NADPH-GDH activity was defined as the amount of enzyme that resulted in one unit of absorbance change (at 340 nm) per minute under the assay condition.

### Intracellular glutamate measurement

The intracellular glutamate concentration was measured using a Glutamic acid Assay Kit (Jian Cheng, China) following the manufacturer’s protocol. The supernatants used for the NADPH-GDH assay were also used for the measurement of the intracellular glutamate concentration.

### Real-time quantitative PCR (qRT-PCR) analysis

Real-time quantitative PCR (qRT-PCR) was performed to compare the *odhA* gene expression levels between wild-type and mutant strains. Cells were harvested for RNA extraction after 16 h of cultivation in LB medium. The total RNA was extracted using the RNApure Bacteria Kit (DNase I) (Cwbio, China). cDNA was extracted using the HiFi-MMLV cDNA Kit (Cwbio, China). RT-qPCR was performed using RealMasterMix (SYBR Green) Kit (Cwbio, China) following the protocol from the manufacturer. The transcription level of *odhA* gene was normalized against that of *rspU* [19]. All the samples were analyzed in five independent experiments. The results were reported as fold changes compared with the control strain.

## Results and discussion

### Effect of NADPH-dependent GDH on γ-PGA production

There are two glutamate biosynthesis pathways existing in nature: GS-GOGAT pathway and the NADPH-dependent GDH pathway (Figs. 1, 2) [20]. GS-GOGAT pathway consists of two steps: in the first step, glutamine was synthesized through incorporating ammonium into glutamate by the ATP-dependent glutamine synthetase (GS). Then in the second step, two molecules of glutamate are generated by transferring the amide group of one molecule of glutamine to one molecule of ɑ-oxoglutarate [catalyzed by the glutamate synthase (GOGAT)] (Fig. 1a). NADPH-dependent GDH pathway forms glutamate through the reductive amination of ɑ-oxoglutarate with the catalysis of GDH (Fig. 1b). Comparing these two pathways, it is apparent that the NADPH-dependent GDH pathway can save one molecule ATP when one molecule glutamate is synthesized. Therefore, it is an energy-saving glutamate synthesis pathway.

Most of the bacteria, such as *B. amyloliquefaciens* used in this study, can only use GS-GOGAT pathway to synthesize glutamate [20], while there are some bacteria able to use both pathways to synthesize glutamate [20–22]. There was evidence that GS–GOGAT pathway is more appropriate to implement in a nitrogen-limited environment, while NAPDH-dependent GDH pathway is only active at high intracellular ammonia concentrations due to its low affinity for ammonium [23]. *C. glutamicum* is able to produce large amount of glutamate and it has long been used for industrial glutamate production [5, 6] *C. glutamicum* has both glutamate production pathways as discussed above; we speculated that its high glutamate production might be due to such unique glutamate synthetic characters. Therefore, we decided to introduce the energy-saving NADPH-dependent glutamate synthesis pathway from *C. glutamicum* into *B. amyloliquefaciens* to increase its native glutamate synthesis capability and thus increase the γ-PGA production.

**Fig. 3:**
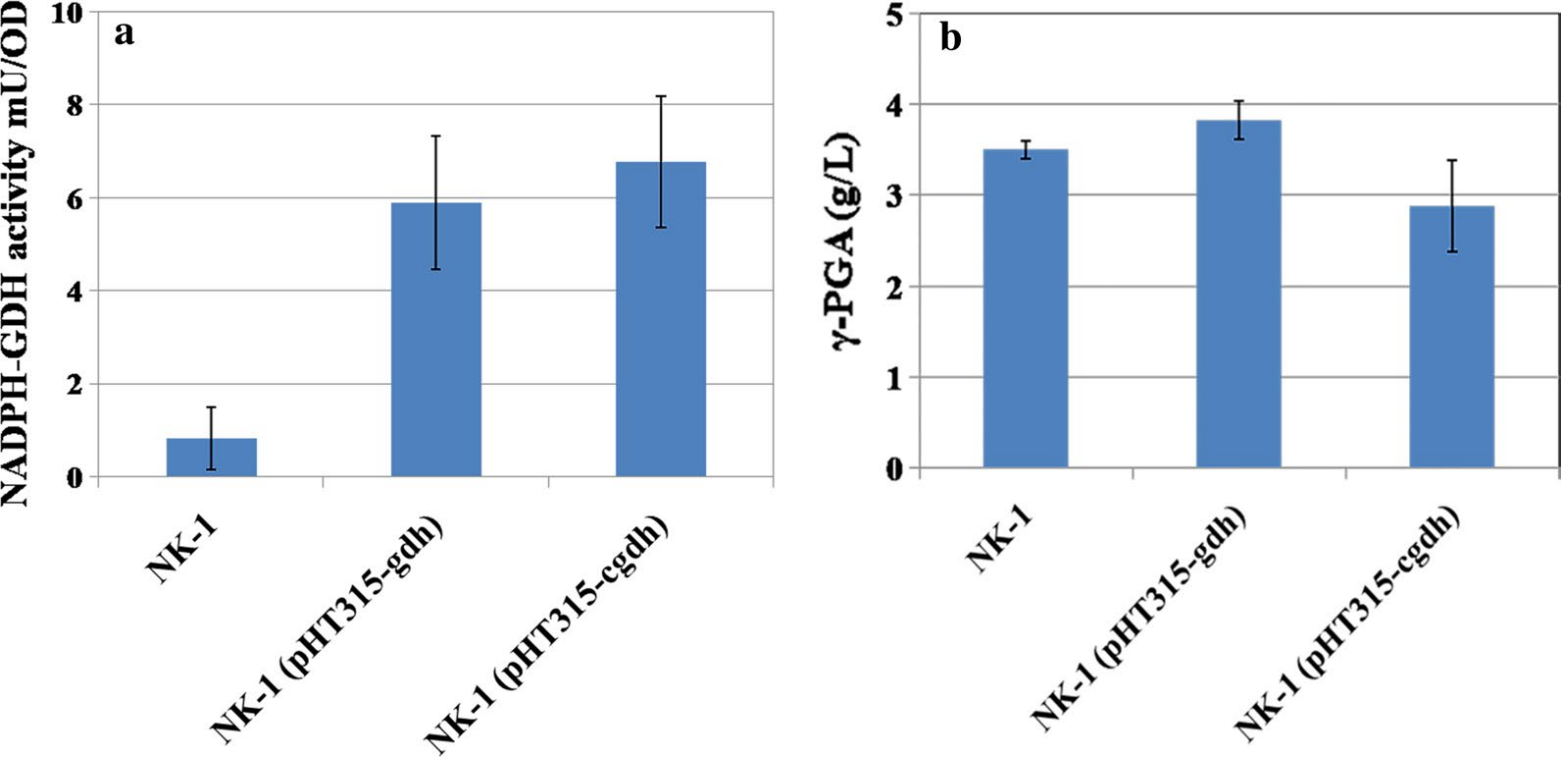
Comparison of the NADPH-GDH activity (**a**) and the γ-PGA production (**b**) among NK-1, NK-1 (pHT315-gdh) and NK-1 (pHT315-cgdh) strains. The reported values represent mean ± SD of triplicates

The native NADPH-dependent glutamate dehydrogenase gene from *C. glutamicum* ATCC13032 (*cgdh*) and the codon optimized *gdh* gene were expressed in NK-1 strain respectively. As shown in Fig. 3a, the NADPH-GDH activities in NK-1 (pHT315-gdh) and NK-1 (pHT315-cgdh) strains were significantly higher than that of the control NK-1 strain. In NK-1 strain, the decrease of NADPH could also be detected, which might be due to the existence of NADPH-quinone reductase. NADPH-quinone reductase can hydrolyze NADPH and thus affected the NADPH-dependent glutamate dehydrogenase measurement results [24]. The intracellular glutamate levels in NK-1 (pHT315-gdh) and NK-1 (pHT315-cgdh) strains were 20.60 and 24.14 μmol/L·OD, respectively; and they were significantly higher than that of the control NK-1 strain (9.48 ± 0.49 μmol/L·OD) (Additional file 3: Table S2). All these results indicated that the heterologous protein functions well in the *Bacillus amyloliquefaciens* strain and increases the intracellular glutamate in the engineered strain. The NADPH-GDH activity and intracellular concentration of glutamate in NK-1 (pHT315-cgdh) strain were all higher than that of the NK-1 (pHT315-gdh) strain, suggesting that the translation level of native gene from *C. glutamicum* ATCC13032 is higher than the codon optimized one. Genetic codon and mRNA structure are two important factors for gene translation. We speculated that the native gene can transcribe into more stable structure of mRNA, and therefore led to higher translation level than the codon optimized gene.

The NK-1 (pHT315-gdh) strain showed 9.1% increase in γ-PGA production, while the NK-1 (pHT315-cgdh) had about 17.8% decrease in γ-PGA production (Fig. 3b). The overexpression of codon optimized *gdh* led to better performance than the overexpression of the origin *cgdh* from *C. glutamicum*. However, the result was not as good as was expected (especially it was a little bit surprising that the overexpression of *cgdh* resulted in decreased γ-PGA production). The strain [NK-1 (pHT315-gdh)] with lower intracellular glutamate concentration showed higher γ-PGA production compared to the NK-1 (pHT315-cgdh) strain. The reason might be that only the increase of the intracellular glutamate within an appropriate range can enhance the target γ-PGA production (in another words, not necessarily the higher the intracellular glutamate level, the higher the γ-PGA production). The extremely higher intracellular glutamate concentration could possibly disrupt the cell metabolic flux balance, and hence inhibit the target product synthesis. The results from the following section also confirmed this hypothesis.

### Construction and verification of the metabolic toggle switch

Besides the existence of two glutamate synthetic pathways, *C. glutamicum* possesses other unique features related to glutamate production. Uy et al. [9] investigated the gene regulation in *C. glutamicum* in continuous culture at different conditions. They found that the activity of ODHC was completely inhibited when glutamate was actively produced. The activity of ODHC seems tightly related to the glutamate production. Rational control of the ODHC expression at specific growth phase is very important for the functionality of the introduced glutamate synthesis feature. Metabolic toggle switch is a novel metabolic engineering strategy for the control of target gene expression as well as the metabolic flux redirection [25]. Therefore, we established this feature in the NK-1 strain by introducing a metabolic toggle switch to evaluate its effect on the γ-PGA production. The metabolic toggle switch can control the expression of ODHC, and hence may affect the glutamate production and further the γ-PGA production.

**Fig. 4:**
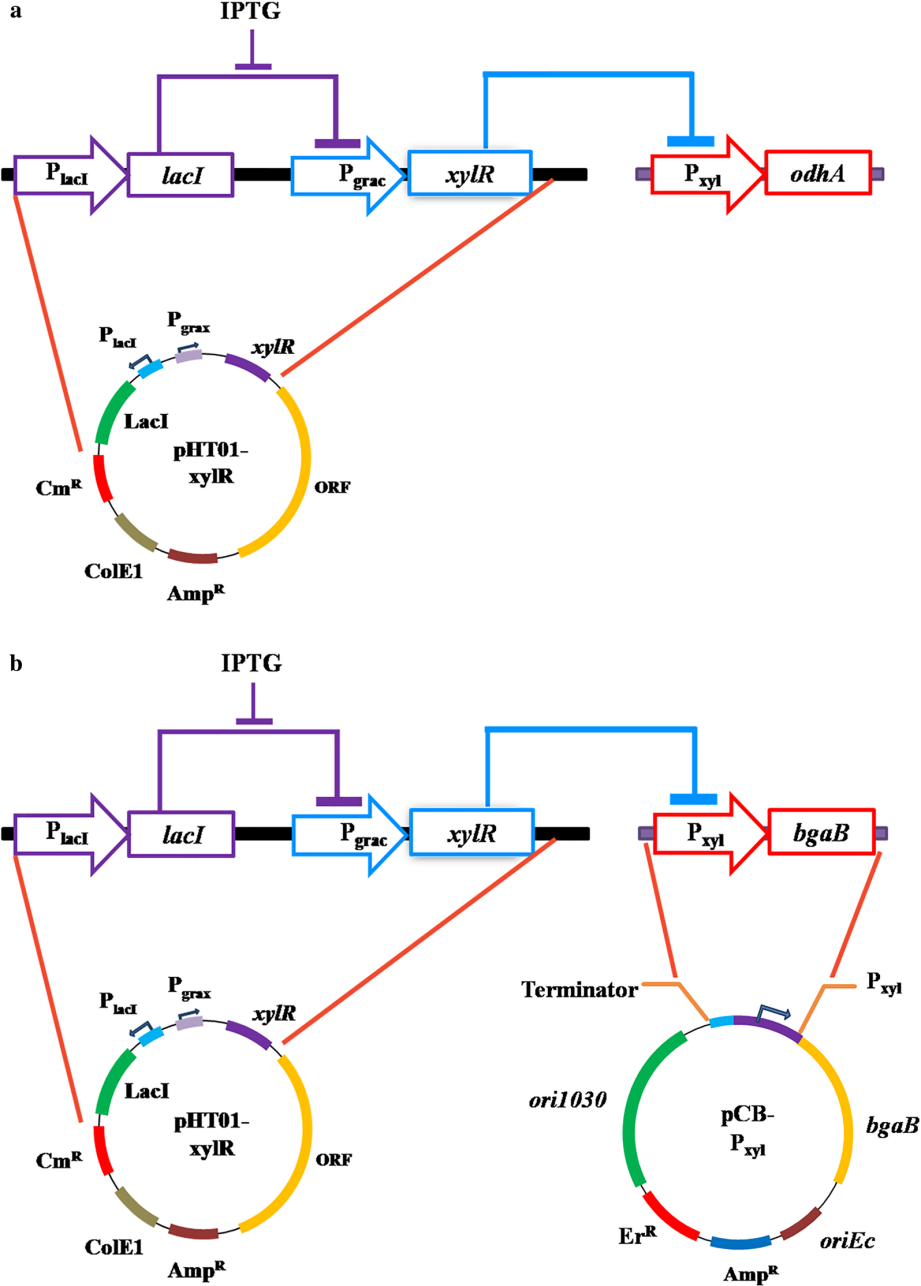
Schematic of the metabolic toggle switch construction. **a** the design of the metabolic toggle switch for controlling *odhA* expression; **b** the schematic design for the verification of the metabolic toggle switch using a *bgaB* reporter gene

Figure 4a illustrated the metabolic toggle switch that we constructed. We firstly inserted a xylose inducible promoter P_xyl_ in the upstream the *odhA* gene. The IPTG inducible *xylR* expression cassette was put on the pHT01 plasmid. In the absence of IPTG, *xylR* expression was repressed and the *odhA* gene normally expressed; in the presence of IPTG, *xylR* gene expressed, leading to the repression for the *odhA* expression. With such strategy, the expression of ODHC can be controlled by the addition of extracellular IPTG.

In order to verify the function of the metabolic toggle switch, we put the P_xyl_ promoter upstream of a reporter *bgaB* gene on the pCB plasmid. The generated pCB-P_xyl_ plasmid was co-expressed with pHT01-xylR in the NK-1 strain (Fig. 4b). The NK-1 (pHT01 + pCB-P_xyl_) strain and NK-1 (pHT01-xylR + pCB-P_xyl_) strain were cultured in the γ-PGA fermentation medium with 1 mM IPTG added in the broth after 12 h of cultivation. In the following 20 h, the β-galactosidase activities in both strains were determined. The results were shown in Additional file 4: Figure S1. Interestingly, the β-galactosidase activity in NK-1 (pHT01-xylR + pCB-P_xyl_) was higher than that in the NK-1 (pHT01 + pCB-P_xyl_) in the first 12 h after addition of IPTG (12–24 h after initiation of the fermentation). However, after 30 h (from the initiation of the fermentation) the β-galactosidase activity in NK-1 (pHT01-xylR + pCB-P_xyl_) became lower than that in NK-1 (pHT01 + pCB-P_xyl_). These results demonstrated that the metabolic toggle switch can function well in NK-1 strain. However, the metabolic toggle switch did not affect the target protein expression immediately after the inducer was added. Possible reasons for such a delay include: (1) the synthesis of XylR needs time after the inhibitor LacI was removed by IPTG addition; (2) the degradation of the generated ODHC prior to the IPTG addition also needs some time to occur. It has been reported that such hysteresis can be mitigated by adding a degradation tag at the end of target protein. Cameron et al. [26] added a Pdt degradation tag at the end of LacI. The LacI-pdt can be recognized by the mf-Lon protease and thus be degraded immediately. Such a design can improve the function of the deigned particular genetic circuit. However, in this study, we didn’t implement this. There might be delay in the functionality of the designed construct in this study, but this did not lead to negative impact on the targeted γ-PGA production improvement. Moreover, ODHC is an important enzyme in the TCA cycle and cells need to maintain its activity during growth. The Pdt tagged ODHC might be able to accelerate the performance of the toggle switch but it would also continuously consume energy for ODHC synthesis at the early stage of the cell growth, which might lead to the decrease of target product. This warrants further investigation in our future work.

### Effects of the metabolic toggle switch on γ-PGA production

After verified the function of the metabolic switch, we further investigated its effect on γ-PGA production in the NK-TP strain, in which the *odhAB* was controlled by the P_xyl_ promoter. In order to determine the optimal time for IPTG addition, fermentations were carried out with 1 mM IPTG was added to each fermentation at different time points (different by 3 h from 0 to 24 h; Additional file 5: Figure S2). The results demonstrated that all the fermentations including the fermentation with NK-TP strain without IPTG addition showed decreased γ-PGA production compared to the fermentation with the NK-1 strain. Then, we decided to test whether higher concentrations of IPTG supplementation can lead to a better repression of *odhA* expression (and thus higher γ-PGA production). We measured the *odhA* expression levels in the NK-TP (pHT01-xylR) strain with IPTG supplementation at various concentrations (1, 5 and 10 mM) comparing to that in the NK-1 strain. The results indicated that the *odhA* expression in NK-TP (pHT01-xylR) strain was generally all tenfold higher than that in the NK-1 strain under all tested conditions (data not shown). This suggested that the *odhA* expression with the leaky activity of P_xyl_ promoter was even higher than the natural *odhA* expression in the NK-1 strain.

**Fig. 5:**
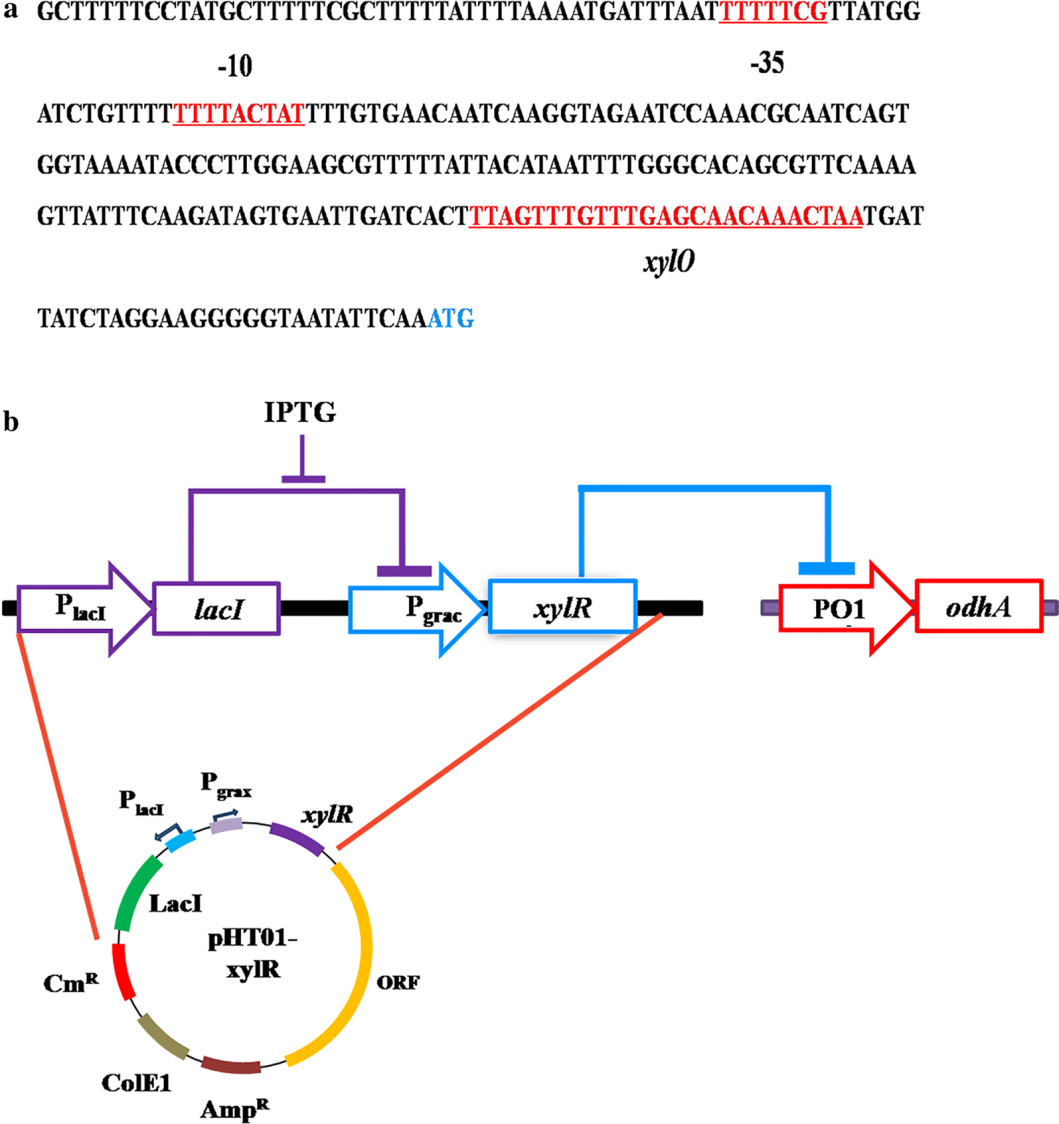
The modified metabolic toggle switch. **a** the design of the XylR regulated PO1 promoter; **b** the schematic design of the modified metabolic toggle switch

To solve this problem, we redesigned the native promoter of the *odhAB* to be a xylose inducible promoter by inserting a *xylO* operator into the native promoter sequence (Fig. 5a). The *xylO* is the xylose repressor (XylR) binding site; by inserting a *xylO* sequence in the promoter transcription region, the promoter will be engineered into a XylR repressed and xylose induced promoter [27, 28]. This promoter engineering strategy is similar with the construction of the IPTG induced promoter P_grac_. By inserting a *lacO* sequence between the *groE* promoter-10 region and SD sequence, the generated promoter P_grac_ could be repressed by LacI and induced by IPTG [29].

**Fig. 6:**
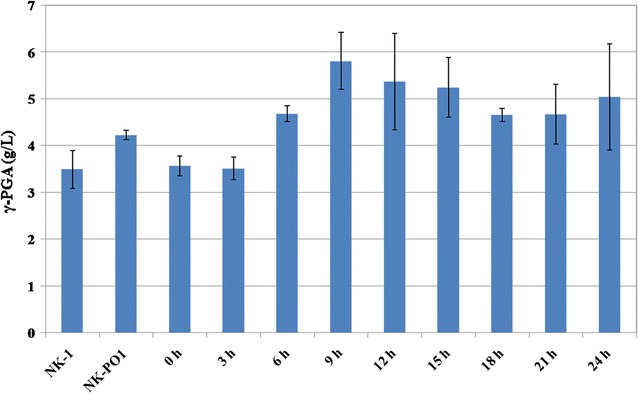
Comparison of γ-PGA production through fermentation with NK-1, NK-PO1 and NK-PO1 (pHT01-xylR) strains. 1 mM IPTG was added into each fermentation at different time point (different by 3 h from 0–24 h of the fermentation). Values represent mean ± SD of triplicates

The generated PO1 promoter was then inserted into the upstream of the *odhA* gene (along with the *spoVG* gene transcription terminator upstream the PO1 promoter to prevent the leaky activity of the natural *odhA* gene promoter) and generated the NK-PO1 strain. The pHT01-xylR plasmid was transformed into the NK-PO1 strain to complete the whole redesigned toggle switch (Fig. 5b). Fermentations were carried out with the NK-PO1 (pHT01-xylR) strain using the γ-PGA fermentation medium for 48 h, with 1 mM IPTG was added to each fermentation at different time points (different by 3 h from 0 to 24 h). As shown in Fig. 6, the γ-PGA production in the NK-PO1 strain (without IPTG addition) was increased by 37.4% compared to the NK-1 strain. The highest γ-PGA production was obtained in the NK-PO1 (pHT01-xylR) strain with the addition of IPTG after 9 h of cultivation (5.81 g/L, which was 66.2% higher than that from the NK-1 strain). The re-designed genetic circuit seemed to function well. However, when IPTG was added at 0 or 3 h, the γ-PGA production was lower than the case without IPTG addition. This might be because the early repression of *odhA* expression resulted in the inhibition of TCA cycle, and thus inhibited the γ-PGA production. We further compared the *odhA* gene transcription levels in NK-1, NK-PO1 and NK-PO1 (pHT01-xylR) (with supplement of 1 mM IPTG at 9 h of cultivation) and found that the *odhA* trancription levels in NK-PO1 and NK-PO1 (pHT01-xylR) strain was about 43.4 and 17.8% of that of the NK-1 strain. The down-regulation of *odhAB* also resulted in the increase of intracellular glutamate level (Additional file 3: Table S2). The intracellular glutamate concentration of NK-PO1 and NK-PO1 (pHT01-xylR) strains were 11.09 and 13.73 μmol/L·OD, both of which were higher than that of the control NK-1 strain (9.48 mol/L·OD). The replacement of native P_odhAB_ promoter by the PO1 promoter down-regulated the *odhAB* transcription and thus enhanced the synthesis of glutamate and γ-PGA. The addition of IPTG after 9 h of fermentation could further down-regulate the *odhAB* transcription (without affecting the regular TCA circle) and enhance the intracellular glutamate and γ-PGA production additionally.

These results demonstrated that the introduction of the metabolic toggle switch (with optimized time point for IPTG supplementation for induction) can rationally control the *odhAB* expression, and therefore lead to the increase of intracellular glutamate concentration as well as the γ-PGA production.

### Combination of two strategies and the effect on γ-PGA production

To further improve the γ-PGA production, we combined the two strategies as discussed above (the NADPH-dependent GDH pathway and the metabolic toggle switch) and generated *B. amyloliquefaciens* NK-PO1 (pHT01-xylR + pHT315-gdh) and *B. amyloliquefaciens* NK-PO1 (pHT01-xylR + pHT315-cgdh). Fermentation was carried out using these strains in different media (γ-PGA fermentation medium and P5 medium [30]), with IPTG supplementation at various time points. However, the results were not as expected. The two strains generated highest γ-PGA titers of 2.90 and 2.82 g/L, respectively, which were at least 16.9% lower than the control. The intracellular glutamate concentrations of NK-PO1 (pHT01-xylR + pHT315-gdh) and NK-PO1 (pHT01-xylR + pHT315-cgdh) strains were 19.42 and 25.60 μmol/L·OD and they were all higher than that of the NK-1 strain and NK-PO1 strain. As was discussed above, the appropriate concentration of intracellular glutamate is very important for the improvement of γ-PGA production. If the intracellular glutamate concentration is extremely high, the expression level of γ-PGA synthase can’t match the increase of intracellular glutamate, which will result in the imbalance of cell metabolic flux and thus inhibit the synthesis of γ-PGA. Besides, we also noticed that the NK-PO1 (pHT01-xylR + pHT315-gdh) and NK-PO1 (pHT01-xylR + pHT315-cgdh) strains had lower cell dry weights of 1.32 and 1.36 g/L, respectively when compared with the control NK-1 strain (1.57 g/L) and NK-PO1 strain (1.48 g/L). The lower cell dry weight might be another reason for the decrease of γ-PGA production. It seems that the use of two plasmids in the same host resulted in a burden for the cell. Base on the above results, couple of strategies can be implemented in the future to optimize the combination of the NADPH-dependent GDH pathway and the metabolic toggle switch for the further improvement of γ-PGA production. Firstly, the intracellular glutamate concentration should be controlled to alleviate the metabolic imbalance caused by the overproduction of intracellular glutamate. Dynamic sensor-regulator system (DSRS) can sense the key intermediate and dynamically regulate the genes involved in the target product synthesis [31]. Therefore, a DSRS system based on the glutamate sensor can be constructed to control intracellular glutamate concentration and dynamically regulate γ-PGA synthesis. Secondly, we can consider to integrate the genetic circuit into the chromosome. The chromosome integration can avoid the use of multiple plasmids and thus eliminate the related metabolic burden.

## Conclusions

In this study, we aimed to improve the γ-PGA production by increasing the intracellular glutamate availability. We integrated the specific glutamate synthesis characters from *C. glutamicum* to the glutamate-independent γ-PGA-producing *B. amyloliquefaciens* NK-1. An energy-saving NADPH-dependent glutamate dehydrogenase pathway was first introduced, and it can function well in the heterologous strain and improved the intracellular glutamate synthesis. However, only the NK-1 (pHT315-gdh) strain with the native *gdh* gene expressed showed slight increase in γ-PGA production than the NK-1 strain; while the NK-1 (pHT315-cgdh) strain with the overexpression of the codon optimized *gdh* showed decreased γ-PGA production. Alternatively, a modified metabolic toggle switch was introduced into the NK-1 strain to control the ODHC expression as it was reported that the ODHC in *C. glutamicum* was completely inhibited when this strain actively produced glutamate. The generated NK-PO1 (pHT01-xylR) strain (with the supplementation of 1 mM IPTG after 9 h of cultivation) produced 66.2% more γ-PGA than the NK-1 strain. Unexpectedly, the combination of the two strategies (introducing both NADPH-GDH pathway and the metabolic toggle switch) did not lead to further increase of γ-PGA production but rather decreased γ-PGA production than that in the NK-1 strain. We concluded that the appropriate intracellular glutamate concentration is very important for the cell metabolism as well as the γ-PGA production. More work need to be done to rationally combine the two strategies in the same host for further enhancement of γ-PGA production.

## Additional files

**Additional file 1: Table S1.** Primers used in this work.

**Additional file 2.** Genes sequences used in this article.

**Additional file 3: Table S2.** Intracellular glutamate concentrations among different strains.

**Additional file 4: Figure S1.** Verification of the function of metabolic toggle switch using the *bgaB* reporter gene.

**Additional file 5: Figure S2.** Comparison of γ-PGA production through fermentation with NK-1, NK-TP and NK-TP (pHT01-xylR) strains.

## Abbreviations

γ-PGA: poly-γ-glutamic acid
ODHC: ɑ-oxoglutarate dehydrogenase complex
GDH: glutamate dehydrogenase
NADPH: nicotinamide adenine dinucleotide phosphate
NAD: nicotinamide adenine dinucleotide
NADPH-GDH: NADPH-dependent glutamate dehydrogenase
GS-GOGAT: glutamine synthetase-glutamate ɑ-oxoglutarate aminotransferase
IPTG: isopropyl-β-d-thiogalactoside
TCA cycle: tricarboxylic acid cycle
ATP: adenosine triphosphate
ADP: adenosine diphosphate
