# Supplementary material for: Enhancing poly-γ-glutamic acid production in Bacillus amyloliquefaciens by introducing the glutamate synthesis features from Corynebacterium glutamicum
Source: Microb Cell Fact. 2017 May 22;16:88. doi: 10.1186/s12934-017-0704-y (PMC5440981; doi:10.1186/s12934-017-0704-y)
Supplement: Supplementary file 1 — Additional file 1: Table S1. Primers used in this work. [file 12934_2017_704_MOESM1_ESM.pdf]

Table S1 Primers used in this work.

Primers	Sequences (5' to 3')
odh-SF	CCCC*GGATCC*ATCGTGTTTTCAGACGGAGAGCC
odh-SR	TTTTAAGAAGCTCGAGTTGAATATTACCCCCAACCTAGATA
Pxyl-F	GGGGGTAATATTCAACTCGAGCTTCTTAAAAAATAACCAA
Pxyl-R	ATTATTTTGAAACATTTGTCATTTCCCCCTTTGATTTAAG
odh-XF	AGGGGGAAATGACAAATGTTTCAAAATAATATGAAACAAAG
odh-XR	CCCC*GTCGAC*TTCGAAATCATGCACATGGTCAAAT
odh-SS	AGACAAATGACGAAACAACTGATGCG
odh-XX	GTTCTCCGGATTCGACCATTTTTGT
Q-odh-F	AATGAATTGGGAAGATTTTTACGGA
Q-odh-R	TGAATCAGATCTGCGGTGACTCGTC
XylR-F	CCCC*GGATCC*GTGGTTATTATTCAAATTGCAGA
XylR-R	CCCC*GGATCC*CTAACTTATAGGGGTAACACTT
cP43-SF	CCCC*TCTAGA*AGCTTCGTGCATGCAGGCCGGGGCAT
cP43-SR	CTCATCAACTGTCATAAGCTTCTGTTATTAATTCTTGTCTGTT
cgdh-SF	ATTAATAACAGAAGCTTATGACAGTTGATGAGCAGGTCTCTAAC
cgdh-XR	CCCC*GAATTC*TTAGATGACGCCCTGTGCCAGCATCG
P43-F	CCCC*TCTAGA*AGCTTCGTGCATGCAGGCCGGGGCAT
gdh-R	CCCC*GAATTC*CCATGCTTGCTCAGGGCGTGATCTAA
PO1-SR	CCCCCTTCCTAGATAATCATTAGTTTGTTGCTCAAACAAACTAAAGTGA TCAATTCACTATCTTGAAA
PO1-XF	CAACAAACTAATGATTATCTAGGAAGGGGGTAATATTCAA**ATG**TTTCAA AAT
