# Supplementary material for: Enhancing poly-γ-glutamic acid production in Bacillus amyloliquefaciens by introducing the glutamate synthesis features from Corynebacterium glutamicum
Source: Microb Cell Fact. 2017 May 22;16:88. doi: 10.1186/s12934-017-0704-y (PMC5440981; doi:10.1186/s12934-017-0704-y)
Supplement: Supplementary file 2 — Additional file 2. Genes sequences used in this article. [file 12934_2017_704_MOESM2_ESM.pdf]

Genes Sequences used in this article:

P_{xyl} promoter

TCCTTTGTTTATCCACCGAACTAAGTTGGTGTTTTTTGAAGCTTGAATTAGA

TATTTAAAAGTATCATATCTAATATTATAACTAAATTTTCTAAAAAAAACATTG

AAATAAACATTTATTTTGTATATGATGAGATAAAGTTAGTTTATTGGATAAAC

AAACTAACTCAATTAAGATAGTTGATGGATAAACTTGTTCACTTAAATCAAA

GGGGGAAATGACAA

PO1 promoter

GCTTTTTCCTATGCTTTTTCGCTTTTTATTTTAAAATGATTTAAT<u>TTTTTCG</u>TTA

TGGATCTGTTTTT<u>TTTTACTAT</u>TTTGTGAACAATCAAGGTAGAATCCAAACGC

AATCAGTGGTAAAATACCCTTGGAAGCGTTTTTATTACATAATTTTGGGCAC

AGCGTTCAAAAGTTATTTCAAGATAGTGAATTGATCACT<u>TTAGTTTGTTTGA</u>

<u>GCAACAAACTAA</u>TGATTATCTAGGAAGGGGGTAATATTCAA

***cgdh* (from *C. glutamicum* ATCC13032)**

ATGACAGTTGATGAGCAGGTCTCTAACTATTACGACATGCTTCTGAAGCGC

AATGCTGGCGAGCCTGAATTTCACCAGGCAGTGGCAGAGGTTTTGGAATCT

TTGAAGATCGTCCTGGAAAAGGACCCTCATTACGCTGATTACGGTCTCATC

CAGCGCCTGTGCGAGCCTGAGCGTCAGCTCATCTTCCGTGTGCCTTGGGTT

GATGACCAGGGCCAGGTCCACGTCAACCGTGGTTTCCGCGTGCAGTTCAA

CTCTGCACTTGGACCATACAAGGGCGGCCTGCGCTTCCACCCATCTGTAAA

CCTGGGCATTGTGAAGTTCCTGGGCTTTGAGCAGATCTTTAAAAACTCCCT

AACCGGCCTGCCAATCGGTGGTGGCAAGGGTGGATCCGACTTCGACCCTA

AGGGCAAGTCCGATCTGGAAATCATGCGTTTCTGCCAGTCCTTCATGACCG

AGCTACACCGCCACATCGGTGAGTACCGCGACGTTCCTGCAGGTGACATCG

GAGTTGGTGGCCGCGAGATCGGTTACCTGTTTGGCCACTACCGTCGCATGG

CTAACCAGCACGAGTCCGGCGTTTTGACCGGTAAGGGCCTGACCTGGGGT

GGATCCCTGGTCCGCACCGAGGCAACTGGCTACGGCTGCGTTTACTTCGTG

AGTGAAATGATCAAGGCTAAGGGCGAGAGCATCAGCGGCCAGAAGATCAT

CGTTTCCGGTTCCGGCAACGTAGCAACCTACGCGATTGAAAAGGCTCAGG

AACTCGGCGCAACCGTTATTGGTTTCTCCGATTCCAGCGGTTGGGTTCATAC

CCCTAACGGCGTTGACGTGGCTAAGCTCCGCGAAATCAAGGAAGTTCGTC

GCGCACGCGTATCCGTGTACGCCGACGAAGTTGAAGGCGCAACCTACCAC

ACCGACGGTTCCATCTGGGATCTCAAGTGCGATATCGCTCTTCCTTGTGCAA

CTCAGAACGAGCTCAACGGCGAGAACGCTAAGACTCTTGCAGACAACGGC

TGCCGTTTCGTTGCTGAAGGCGCGAACATGCCTTCCACCCCTGAGGCTGTT

GAGGTCTTCCGTGAGCGCGACATCCGCTTCGGACCAGGCAAGGCAGCTAA

CGCTGGTGGCGTTGCAACCTCCGCTCTGGAGATGCAGCAGAACGCTTCGC

GCGATTCCTGGAGCTTCGAGTACACCGACGAGCGCCTCCAGGTGATCATGA

AGAACATCTTCAAGACCTGTGCAGAGACCGCAGCAGAGTATGGACACGAG

AACGATTACGTTGTCGGCGCTAACATTGCTGGCTTCAAGAAGGTAGCTGAC

GCGATGCTGGCACAGGGCGTCATCTAA

***gdh* (codon optimized cgdh gene)**

ATGACAGTTGATGAACAAGTGTCTAACTATTACGACATGCTGCTTAAAAGA

AATGCCGGAGAACCGGAATTTCATCAGGCAGTCGCGGAAGTATTAGAATCC

TTGAAAATCGTGTTAGAAAAAGATCCTCATTACGCAGACTACGGCTTAATTC

AACGCTTGTGCGAACCGGAAAGACAGTTGATCTTTCGCGTTCCTTGGGTGG

ATGACCAAGGCCAGGTCCATGTAAACCGTGGATTTCGGGTCCAATTTAATT

CTGCGTTAGGACCGTATAAAGGCGGATTGCGTTTTCATCCTTCCGTCAACTT

GGGAATCGTAAAATTTTTGGGTTTTGAACAGATCTTTAAAAACTCTCTGAC

AGGACTTCCGATTGGTGGCGGAAAAGGTGGCTCAGATTTTGACCCTAAAG

GCAAAAGCGATTTAGAAATCATGCGTTTTTGCCAATCATTTATGACGGAATT

GCATCGTCATATCGGAGAATATCGGGATGTCCCGGCGGGCGACATTGGAGT

AGGAGGTCGGGAAATCGGTTATTTATTTGGCCATTACAGACGCATGGCTAA

CCAGCATGAATCAGGCGTCCTGACAGGAAAAGGTCTTACGTGGGGCGGAA

GCCTTGTAAGAACAGAAGCCACGGGCTATGGATGTGTCTACTTTGTAAGCG

AAATGATCAAAGCAAAAGGAGAATCTATCTCCGGTCAAAAAATTATCGTTT

CAGGTAGCGGCAACGTGGCAACATATGCGATTGAAAAAGCACAGGAACTG

GGCGCGACGGTCATCGGATTTTCTGATTCAAGCGGTTGGGTACATACACCG

AATGGCGTTGACGTGGCCAAACTTAGAGAAATTAAAGAAGTTCGTCGGGC

TCGCGTCTCAGTATATGCCGATGAAGTGGAAGGAGCAACATACCATACGGA

CGGCAGCATTTGGGATCTGAAATGCGACATCGCACTTCCTTGTGCGACACA

AAATGAACTGAACGGCGAAAATGCTAAAACGCTTGCCGATAACGGATGCA

GATTTGTTGCTGAAGGCGCCAATATGCCGTCAACACCTGAAGCAGTTGAAG

TGTTTAGAGAACGCGATATCCGCTTTGGACCGGGTAAAGCAGCGAACGCG

GGTGGCGTCGCAACATCTGCGCTGGAAATGCAACAGAATGCTTCCCGTGAT

TCTTGGTCCTTTGAATATACGGACGAACGGCTTCAAGTTATCATGAAAAACA

TCTTTAAAACATGTGCGGAAACGGCTGCCGAATATGGACATGAAAACGATT

ACGTTGTGGGTGCTAATATTGCCGGCTTTAAAAAAGTTGCTGATGCCATGCT

TGCTCAGGGCGTGATCTAA

xylR

GTGGTTATTATTCAAATTGCAGATCAAGCTTTAGTAAAAAAAATGAATCAAA

AATTAATATTAGATGAAATTTTGAAGAACTCCCCTGTCTCCAGGGCAACTCT

CTCTGAGATTACAGGATTAAACAAGTCTACTGTCTCCTCTCAAGTAAATACA

CTGCTTGAAAAAGATTTTATTTTTGAAATTGGGGCAGGGCAATCTAGAGGC

GGCAGAAGACCTGTAATGCTTGTTTTTAATAAGAATGCAGGCTACTCGATTG

GTATTGATATAGGAGTCGACTATCTTAACGGAATTCTAACCGACTTAGAAGG

AAATATTATTCTCGAGAAGACTTCTGACTTGTCTAGTTCTTCCGCTAGTGAA

GTAAAAGAGATTTTATTTGCACTTATTCATGGTTTTGTAACCCATATGCCTGA

GTCCCCTTATGGTCTAGTCGGAATAGGAATTTGTGTTCCAGGCCTTGTAGAT

CGTCATCAGCAAATTATTTTCATGCCTAACTTAAATTGGAATATCAAAGATTT

GCAGTTTTTAATTGAGAGTGAGTTTAATGTTCCGGTTTTTGTTGAAAATGAA

GCTAATGCAGGAGCATACGGTGAAAAAGTATTTGGTATGACAAAAAACTAT

GAAAACATCGTTTACATCAGTATTAATATCGGAATTGGAACTGGACTTGTTA

TTAACAACGAATTGTATAAAGGTGTTCAGGGTTTTTCTGGGGAAATGGGTC

ATATGACGATAGATTTTAATGGACCCAAATGCAGCTGTGGAAATCGAGGCT

GTTGGGAATTATATGCTTCTGAAAAAGCGTTACTGGCTTCGCTCTCTAAAGA

AGAAAAGAATATTTCTCGAAAAGAGATTGTGGAACGCGCAAATAAAAATG

ATGTAGAAATGTTAAATGCACTTCAAAACTTTGGCTTTTATATCGGAATTGG

ATTAACCAATATCCTTAATACATTTGATATAGAAGCTGTTATCTTGAGAAATC

ATATAATTGAATCTCATCCCATTGTTTTAAATACGATTAAAAACGAAGTTTCT

TCTAGAGTCCATTCTCATTTAGACAATAAATGTGAACTATTGCCTTCTTCGTT

AGGAAAAAATGCACCTGCTTTAGGAGCGGTTTCTATCGTTATTGATTCTTTT

TTAAGTGTTACCCCTATAAGTTAG

***spoVG* terminator (from *B. subtilis*)**

CTCGAGCTTCTTAAAAAATAACCAAAAAGCAAGGACTGCTGAAAGGGCTGACATAAG

CCTTTTGCCGGCGGTCCTTTTTTAATTCTGAT
