# Supplementary material for: Enhancing poly-γ-glutamic acid production in Bacillus amyloliquefaciens by introducing the glutamate synthesis features from Corynebacterium glutamicum
Source: Microb Cell Fact. 2017 May 22;16:88. doi: 10.1186/s12934-017-0704-y (PMC5440981; doi:10.1186/s12934-017-0704-y)
Supplement: Supplementary file 3 — Additional file 3: Table S2. Intracellular glutamate concentrations among different strains. [file 12934_2017_704_MOESM3_ESM.pdf]

Table S2 Intracellular glutamate concentrations among different strains

Strain	Glutamate concentration (μmol/L.OD)
B. amyloliquefaciens NK-1	9.48±0.49
B. amyloliquefaciens NK-1 (pHT315-gdh)	20.60±4.15
B. amyloliquefaciens NK-1 (pHT315-cgdh)	24.14±1.37
B. amyloliquefaciens NK-PO1	11.09±0.54
B. amyloliquefaciens NK-PO1 (pHT01-xylR)[#]	13.73±0.68
B. amyloliquefaciens NK-PO1 (pHT01-xylR+pHT315-gdh)[#]	19.42±0.91
B. amyloliquefaciens NK-PO1 (pHT01-xylR+pHT315-cgdh)[#]	25.60±0.75

[#]1 mM IPTG was added into the medium after 9 h of cultivation.
