# Supplementary material for: Enhancing poly-γ-glutamic acid production in Bacillus amyloliquefaciens by introducing the glutamate synthesis features from Corynebacterium glutamicum
Source: Microb Cell Fact. 2017 May 22;16:88. doi: 10.1186/s12934-017-0704-y (PMC5440981; doi:10.1186/s12934-017-0704-y)
Supplement: Supplementary file 4 — Additional file 4: Figure S1. Verification of the function of metabolic toggle switch using the bgaB reporter gene. [file 12934_2017_704_MOESM4_ESM.pdf]

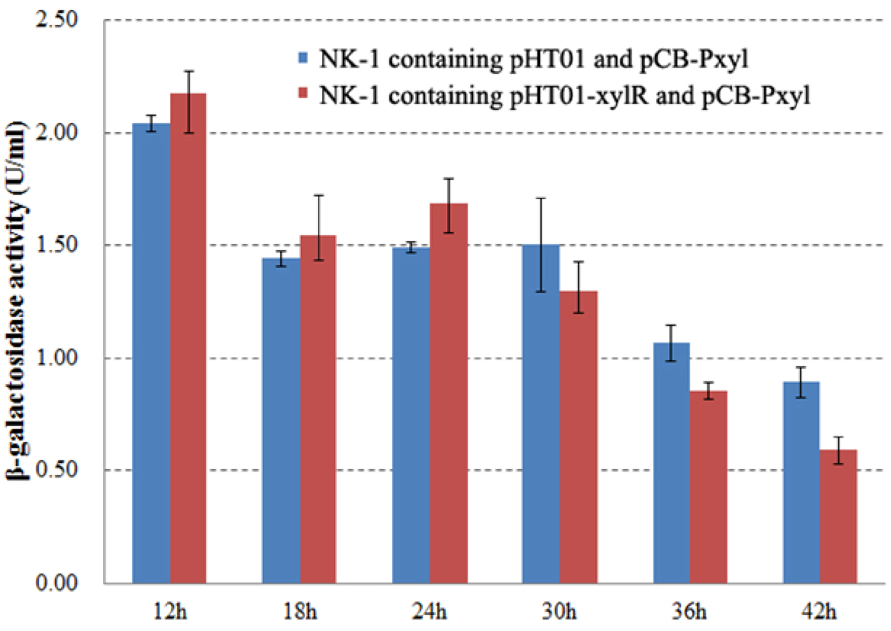

Fig.S1 Verification of the function of metabolic toggle switch using the *bgaB* reporter gene.The NK-1 (pHT01+pCB-P_{xyl}) strain and NK-1 (pHT01-xylR+ pCB-P_{xyl}) strain were cultured at γ-PGA fermentation medium with 1mM IPTG added after 12 h of cultivation. Values represent means ± SD of triplicates.
