# Supplementary material for: Enhancing poly-γ-glutamic acid production in Bacillus amyloliquefaciens by introducing the glutamate synthesis features from Corynebacterium glutamicum
Source: Microb Cell Fact. 2017 May 22;16:88. doi: 10.1186/s12934-017-0704-y (PMC5440981; doi:10.1186/s12934-017-0704-y)
Supplement: Supplementary file 5 — Additional file 5: Figure S2. Comparison of γ-PGA production through fermentation with NK-1, NK-TP and NK-TP (pHT01-xylR) strains. [file 12934_2017_704_MOESM5_ESM.pdf]

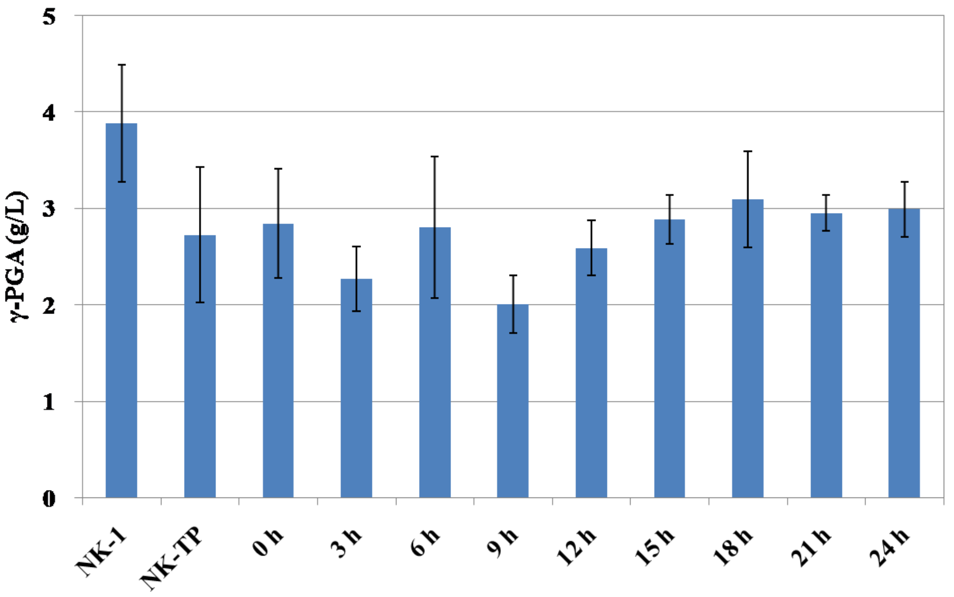

Fig.S2 Comparison of γ-PGA production through fermentation with NK-1, NK-TP and NK-TP (pHT01-xylR) strains.1mM IPTG was added into each fermentation at different time point (different by 3 h from 0 h-24 h of the fermentation). Values represent means ± SD of triplicates.
